# Corrigendum: PD-1/PD-L1 immune checkpoint therapy demonstrates favorable safety profile in patients with autoimmune and cholestatic liver disease

**DOI:** 10.3389/fimmu.2024.1369747

**Published:** 2024-01-24

**Authors:** Lorenz Kocheise, Ignazio Piseddu, Joscha Vonderlin, Eric T. Tjwa, Gustav Buescher, Lucy Meunier, Pia Goeggelmann, Francesca Fianchi, Jérôme Dumortier, Mar Riveiro Barciela, Tom J. G. Gevers, Benedetta Terziroli Beretta-Piccoli, Maria-Carlota Londoño, Sona Frankova, Thomas Roesner, Vincent Joerg, Constantin Schmidt, Fabian Glaser, Jan P. Sutter, Thorben W. Fründt, Ansgar W. Lohse, Samuel Huber, Johann von Felden, Marcial Sebode, Kornelius Schulze

**Affiliations:** ^1^I. Department of Medicine, University Medical Center Hamburg-Eppendorf, Hamburg, Germany; ^2^European Reference Network on Hepatological Diseases (ERN RARE-LIVER), Hamburg, Germany; ^3^Department of Medicine II, University Hospital, Ludwig-Maximilians-Universität (LMU) München, Munich, Germany; ^4^Department of Hepatology and Gastroenterology, Charité-Universitätsmedizin Berlin, Berlin, Germany; ^5^Department of Gastroenterology and Hepatology, Radboud Institute for Molecular Life Sciences, Radboud University Medical Center, Nijmegen, Netherlands; ^6^Service Hépato-Gastro Entérologie, Hôpital St-Eloi, CHU Montpellier, Montpellier, France; ^7^Department of Internal Medicine I, University Hospital Regensburg, Regensburg, Germany; ^8^CEMAD-Centro Malattie dell’Apparato Digerente, Fondazione Policlinico Universitario Gemelli IRCCS, Università Cattolica del Sacro Cuore, Roma, Italy; ^9^Service d’hépato-gastroentérologie, Hôpital Edouard Herriot – Hospices civils de Lyon, Université de Lyon, Lyon, France; ^10^Liver Unit, Department of Internal Medicine, Hospital Universitari Valle d’Hebron, Universitat Autònoma de Barcelona, Barcelona, Spain; ^11^Department of Gastroenterology and Hepatology, Maastricht University Medical Centre, Maastricht, Netherlands; ^12^Nutrim School for Nutrition and Translational Research in Metabolism, Maastricht University, Maastricht, Netherlands; ^13^Epatocentro Ticino, Lugano, Switzerland; ^14^Faculty of Biomedical Sciences, Università della Svizzera Italiana, Lugano, Switzerland; ^15^MowatLabs, Faculty of Life Sciences & Medicine, King’s College London, King’s College Hospital, London, United Kingdom; ^16^Liver Unit, Hospital Clinic Barcelona, FCRB-IDIBAPS, CIBEREHD, University of Barcelona, Barcelona, Spain; ^17^Department of Hepatogastroenterology, Institute for Clinical and Experimental Medicine, Prague, Czechia; ^18^Department of Medical Oncology, National Center of Tumor Diseases (NCT) Heidelberg and Universitätsklinikum Heidelberg, Heidelberg, Germany

**Keywords:** autoimmune disease (AID), immune checkpoint inhibitors (ICI), autoimmune liver diseases (AILD), immune related adverse effects (irAEs), PD-1/PD-L1 immune checkpoint inhibitors, autoimmune hepatitis (AIH), primary sclerosing cholangites (PSC), primary biliary cholangitis (PBC)

In the published article, there was an error in **Table 2**. The count of monoclonal antibodies used was interchanged during production between the different antibodies. The corrected **Table 2** and its caption appear below.

**Table 2:** Immune-related adverse events and immunosuppression.

*Baseline characteristics*	*Patients, n = 22 (%)*
irAE	
Grade 1	3 (13.6)
Grade 2	5 (22.7)
None	14 (63.6)
Liver irAE	
Grade 1	2 (9.1)
Grade 2	1 (4.5)
Non–Liver irAE	
Colitis	1 (4.5)
Pneumonitis	1 (4.5)
Inflammatory arthritis	1 (4.5)
Rash	2 (9.1)
Treatment regimen before ICI^#^	
Mycophenolate mofetil	1 (4.5)
Azathioprine	4 (18.1)
Corticosteroids	2 (9.1)
Methotrexate*	1 (4.5)
UDCA	9 (40.9)
Obeticholic acid	1 (4.5)
No treatment for AILD	7 (31.8)
Treatment adjustment during ICI therapy due to non-liver related events^#^	
Start Hydroxycholoroquine*	1 (4.5)
Start or increase of corticosteroids	2 (9.1)
No change in treatment	16 (72.7)
Treatment adjustment during ICI therapy due to ILICI or AILD^#^	
Start or increase of corticosteroids	2 (9.1)
Start UDCA	1 (4.5)
Change from obeticholic acid to Fenofibrate	1 (4.5)
Stop Azathioprine	1 (4.5)
No change in treatment	16 (72.7)
Monoclonal antibody used	
Atezolizumab	7 (31.8)
Durvalumab	5 (22.7)
Pembrolizumab	4 (18.1)
Nivolumab	4 (18.1)
Nivolumab + Ipilimumab	1 (4.5)
Spartalizumab	1 (4.5)

No changes in immunosuppressive therapy were made before commencing ICI treatment to prevent potential irAEs. #Multiple treatments possible *Treatment for Rheumatoid arthritis.

The authors apologize for this error introduced during production and state that this does not change the scientific conclusions of the article in any way. The original article has been updated.

